# Inequalities in type 2 diabetes incidence in a multiethnic population: a cohort study investigating the impact of ethnicity, migration and mental health comorbidities

**DOI:** 10.1007/s00125-026-06740-3

**Published:** 2026-04-22

**Authors:** Diana Shamsutdinova, Daniel Stahl, Jayati Das-Munshi

**Affiliations:** 1 Department of Biostatistics and Health Informatics, Institute of Psychiatry, Psychology & Neuroscience, King’s College London, London, UK; 2 NIHR Maudsley Biomedical Research Centre, London, UK; 3 South London & Maudsley NHS Trust, London, UK; 4 Department of Psychological Medicine, Institute of Psychiatry, Psychology & Neuroscience, King’s College London, London, UK; 5 ESRC Centre for Society and Mental Health, King’s College London, London, UK; 6 Population Health Improvement UK (PHI UK), London, UK

**Keywords:** Inequality in health outcomes, Severe mental illness, Survival analysis, Type 2 diabetes

## Abstract

**Aims/hypothesis:**

Ethnic disparities in the incidence of type 2 diabetes mellitus are well documented in multiethnic urban populations, but the contributions of migration status and mental health are less well understood. This study used a large dataset from primary care centres in South London that is unique in that it includes migration-related information together with information on mental and physical health comorbidities. We aimed to assess how migration status and mental health contribute to longitudinal associations of ethnicity and type 2 diabetes risk in a multiethnic urban population.

**Methods:**

We conducted a longitudinal cohort study (2012–2019) of approximately 340,000 adults without baseline type 2 diabetes. Cox proportional hazards models were applied with sequential adjustments: first for age and sex; second, adding migration status (country of birth being UK or not); and third, further adding mental health conditions (depression, anxiety, severe mental illness), physical health factors (BMI, hypertension and other macrovascular diseases) and area-level deprivation. This approach allowed us to examine whether ethnic differences in the incidence of type 2 diabetes persist after accounting for additional factors.

**Results:**

South Asian, Black African and Black Caribbean groups had 2–3-fold higher type 2 diabetes risks compared with White British individuals, which were only partially explained by socioeconomic and clinical factors. Being born outside the UK increased type 2 diabetes risk by 29% across all ethnic groups. Depression/anxiety and severe mental illness were associated with a higher risk of type 2 diabetes. No statistical evidence of strong interactions between these factors was obtained.

**Conclusions/interpretation:**

Ethnicity, migration status and mental health conditions were each independently associated with type 2 diabetes risk, and ethnic disparities persisted after adjustment. The lack of evidence for interactions suggests that migration- and mental health-related mechanisms may operate similarly across ethnic groups rather than amplifying or mitigating existing disparities in type 2 diabetes rates. Efforts to reduce diabetes inequalities will require both support for post‑migration challenges and addressing of the broader structural and environmental determinants underlying persistent ethnic disparities.

**Graphical Abstract:**

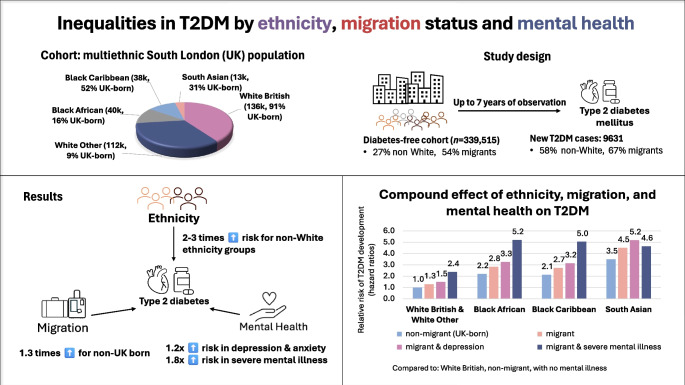

**Supplementary Information:**

The online version contains peer-reviewed but unedited supplementary material available at 10.1007/s00125-026-06740-3.


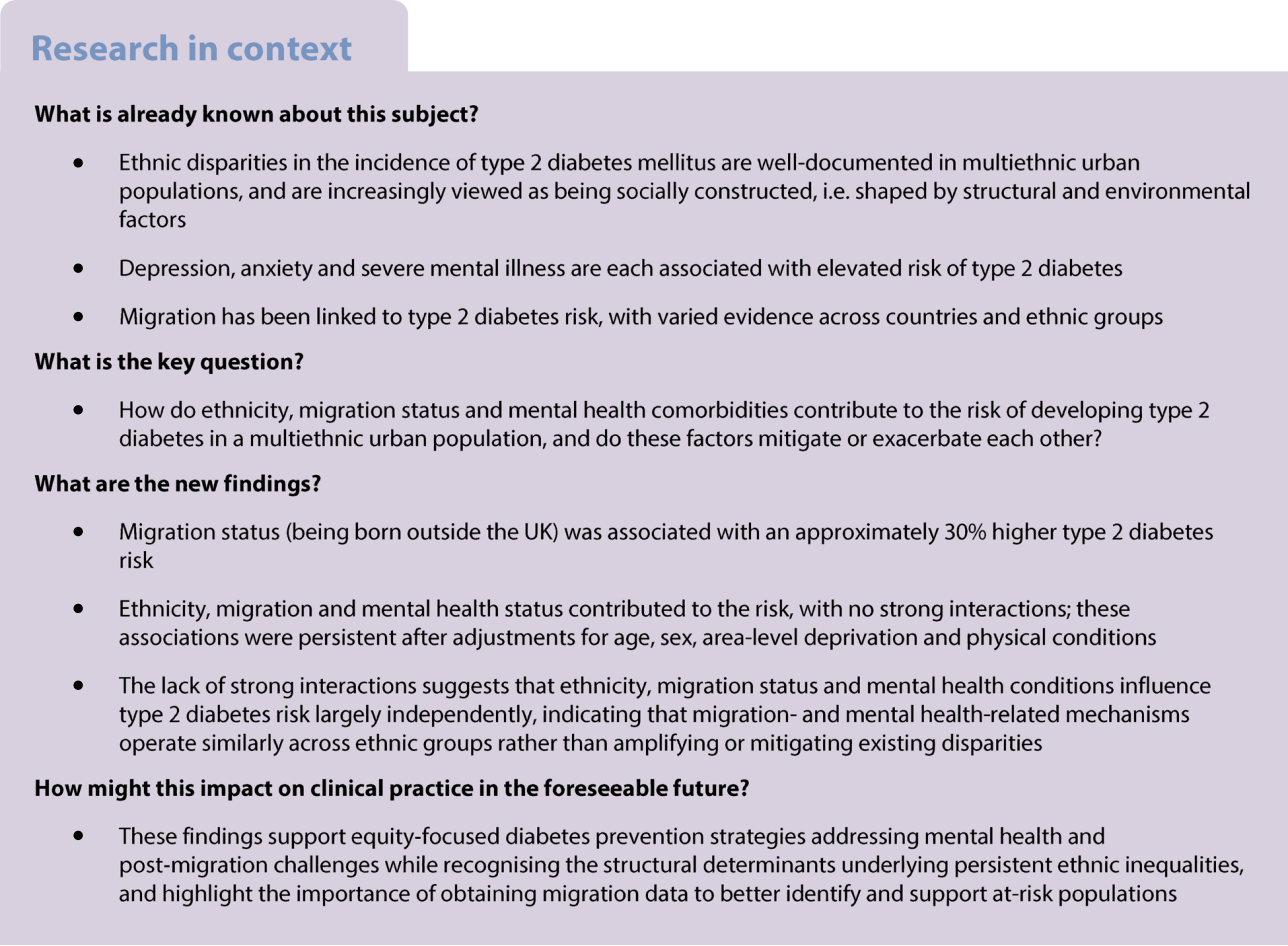


## Introduction

Diabetes is a chronic disorder that is characterised by a persistently elevated blood glucose level. Living with diabetes is associated with a poorer quality of life and lower life expectancy [1]. Diabetes has emerged as a major public health issue, affecting 11% of adults aged 20–79 years globally, and 9% in the UK, with the total medical costs of diabetes management being as high as 2% of gross domestic product in high-income countries [2]. Risk factors for type 2 diabetes include older age, lower socioeconomic status, higher BMI, a family history of diabetes, hypertension, cardiovascular diseases, depression and anxiety, and severe mental illness (SMI) [3].

The prevalence of type 2 diabetes varies considerably by ethnicity, even after adjusting for sociodemographic and physical health exposures. Studies of London’s diverse populations have reported disparities in the incidence and severity [4, 5]; similar patterns have been observed in multiethnic urban settings in Canada [6], Australia [7] and the USA [8–10]. Factors that are thought to contribute to such disparities include living in socially deprived areas, dietary and exercise habits, and potential barriers to healthcare such as language and cultural differences [11].

Migration has been shown to affect type 2 diabetes rates [12, 13], potentially due to the socioeconomic costs of relocation, as well as psychological stresses of migration and living in a foreign country [6, 14]. As ethnic groups vary in the proportion of people with migration experiences, migration may partly explain disparities between the groups. However, findings in this area have been mixed: some studies support the ‘healthy migrant’ phenomenon, whereby immigrants initially exhibit better health outcomes than residents [15–17]; others have reported significantly higher rates of type 2 diabetes and cardiovascular diseases in non-majority ethnic groups, especially among people of South Asian ethnicity [8, 18, 19]. Migration-related health disparities have been studied from various perspectives. For example, migrants have been compared with locally born populations [6, 14], attributing differences primarily to migration experiences. Other studies have examined people of the same origin who have settled in different countries [6], or have compared health outcomes before and after migration [20]. However, only few studies have investigated the interaction of ethnicity and migration status. This could be done, for example, by contrasting first- and second-generation migrants to disentangle the effect of migration-related risk from socio-environmental exposures in the country of residence, as has been done previously for mortality risk [17]. The gap in granular studies of the impact of migration within ethnic groups is partly explained by the difficulties in collecting relevant information, as migration information is not routinely collected in most electronic health records (EHRs).

Mental health conditions have also been linked with type 2 diabetes development. Common mental disorders such as depression and anxiety, as well as SMIs including schizophrenia and bipolar disorder, are associated with an elevated type 2 diabetes risk [21–24]. In the context of migration, relocation stress and adverse pre-migration experiences, especially for forced migration, may act as triggers of mental health conditions [12]. Post-migration experiences, such as acculturation stress due to language and cultural differences, may further exacerbate this relationship [13]. For example, psychosocial stress has been shown to be adversely associated with type 2 diabetes among migrants in the Netherlands, with variations across ethnic groups [25]. However, despite evidence linking both migration and mental health to type 2 diabetes, these factors are rarely examined together or within specific ethnic groups. Much of the current literature considers ethnicity, migration and mental health in isolation, making it difficult to determine how their combined influence contributes to observed disparities in type 2 diabetes incidence.

**Fig. 1:**
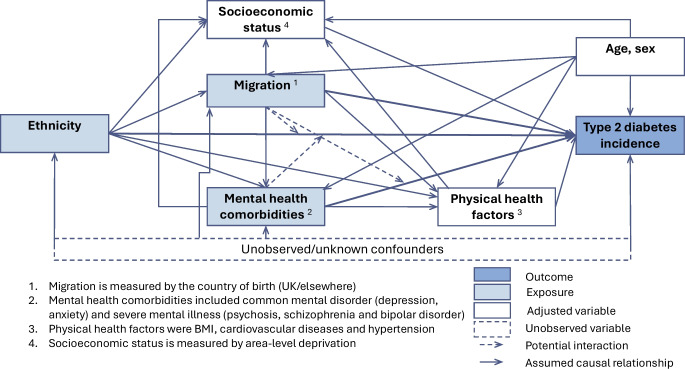
Directed acyclic graph of the hypothesised relationship between ethnicity, migration, mental health and type 2 diabetes

In this study, we leveraged a large longitudinal dataset from a multiethnic population in South London, covering approximately 340,000 individuals observed between 2012 and 2019 (the most recent pre-COVID period, thus avoiding pandemic-related disruptions to help-seeking and EHR recording). These data are unique in containing information on ethnicity (based on UK census classifications [26]) and migration status (whether born outside the UK or not), allowing for a granular analysis of socio-environmental factors contributing to type 2 diabetes. Our aim was to assess the combined and independent contributions of ethnicity, migration status and mental health to inequalities in type 2 diabetes risk. Specifically, we hypothesised that migration status and mental health conditions could partially explain ethnic inequalities in type 2 diabetes risk, either through interacting effects across ethnic groups or by accounting for underlying variation in migration‑related experiences, as shown in Fig. 1 [13, 27].

## Methods

### Cohort

**Fig. 2:**
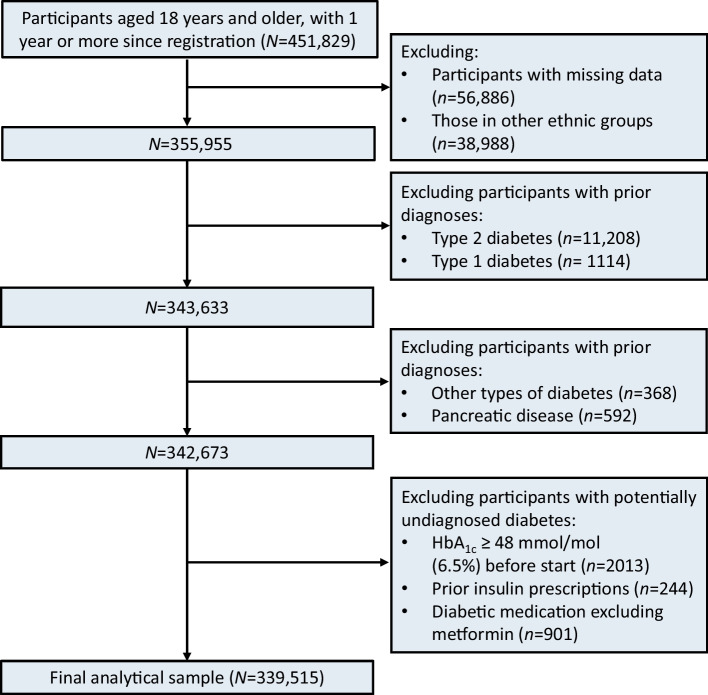
Analytical cohort flowchart

The cohort included people with at least 12 months of consecutive registration with participating primary care centres in South London between 1 April 2012 (study start) and 31 October 2019 (study end), aged $\ge$18 years at study entry, and with no prior type 2 diabetes. The study entry date for a participant was the latest of (1) study start; (2) 12 months after registering with a primary care centre; or (3) reaching 18 years old. The individual end date was the earliest of (1) incident type 2 diabetes diagnosis; (2) leaving the primary care centre; (3) death; or (4) study end. We excluded participants with a history of type 1 diabetes mellitus or other diabetes types (but not diabetes in pregnancy), pancreatic surgery, and those receiving prescriptions for glucose-lowering medications other than metformin, as described in the flowchart shown in Fig. 2.

### Diabetes outcome

Our analytical cohort included participants with no prior diabetes to enable analysis of incident cases.

Prevalent type 2 diabetes cases were established by the presence of either (1) clinical codes for type 2 diabetes; (2) prescribed non-metformin oral glucose-lowering medication: or (3) HbA_1c_ >48 mmol/mol (6.5%) or GT >11.1 mmol/l in the year before joining the study. This algorithm is similar to that described previously [5], whereby type 2 diabetes was identified by clinical codes, then by use of glucose-lowering medications, then by blood glucose measures, and finally by diabetes care codes. The last step only added a small number of additional type 2 diabetes cases (0.5% of the total) in the previous study [5], and was ignored here as we lacked relevant data. Type 1 diabetes was established by either a relevant clinical code or a history of insulin prescription with no non-metformin oral glucose-lowering drugs. Other diabetes types were established by clinical codes, as listed in electronic supplementary material (ESM) Table 1.

Incident type 2 diabetes cases were identified by clinical codes and/or HbA_1c_ readings >48 mmol/mol. For the participants with observed type 2 diabetes incidence, the binary outcome was 1, and participants were referred to as ‘cases’, otherwise the outcome was 0 and the participants were coded as ‘censored’.

### Exposure and covariates

All exposures and covariates were assessed at baseline. Age (years) was entered as a continuous variable, and sex (0, men; 1, women) was coded as a binary variable.

#### Ethnicity (exposure)

Ethnicity was conceptualised as a socially constructed demographic category based on UK census classifications, rather than an indicator of genetic ancestry. The original ethnicity information was collected from participants using the 19 response options from the 2011 UK Census [26], and then re-coded into aggregated categories (ESM Table 2): ‘White British’, ‘White Other’, ‘South Asian’, ‘Other Asian’, ‘Black Caribbean’, ‘Black African’, ‘Other Ethnic Group’ and ‘not reported’, as defined in the 2011 UK Census [26]. The ‘White Other’ category captured people who identified as White but not as White British. We then excluded participants in the heterogeneous ‘Other Asian’ and ‘Other Ethnic Group’ categories from the analyses, as well as those with missing and unreported ethnicity.

#### Migration status

The migration status (or country of birth) variable was coded as 0 if the country of birth was the UK and 1 otherwise.

#### Area-based deprivation index

The area-based deprivation index was determined based on the participants’ postcodes, which were linked to the Lower Layer Super Output Areas defined by the Office of National Statistics [28], and then to the 2015 English Index of Multiple Deprivation (IMD) [29], which aggregates area-level income, education, crime, health and disability levels, resulting in a score that is categorised into ten deciles for the regression analysis, with 1 indicating the most deprived area. For cohort description, deprivation quintiles were used instead.

#### Mental health comorbidities

SMIs comprised diagnosed schizophrenia, bipolar disorder and non-organic psychoses, and were represented by a binary variable that was set to 1 if any of the diagnoses were recorded before the baseline. Diagnosed depression and anxiety were combined into one variable to indicate prevalent common mental illness, equal to 1 if either was diagnosed before the baseline.

#### Physical health comorbidities

The number of prevalent macrovascular diseases excluding hypertension was entered as a continuous variable (0–10). These diseases were ischaemic heart disease, heart failure, stroke, angina, myocardial infarction, atherosclerosis, cardiac valve disease, thrombosis, cardiomyopathy, peripheral vascularisation, peripheral arterial disease and aneurysm, similar to a previous study [4]. Family history of diabetes and hypertension were entered as binary variables (1 or 0). The baseline BMI (kg/m^2^, continuous variable) was the most recent measurement in the year preceding the baseline. All mental and physical health diagnoses were identified by the relevant UK clinical diagnostic codes listed in ESM Table 1 [30].

### Missing values handling

Values were missing for some data: deprivation was missing for 3%, country of birth for 35%, BMI for 31%, HbA_1c_ for 58% (ESM Table 3). To adjust confidence intervals to the presence of missing data, we performed multiple imputations by chained equations (*m*=20) [31]. The results were pooled using Rubin’s rule [32]. Several variables were used in imputation, but not in the main analyses: HbA_1c_ (%), primary language spoken (0 for English, 1 otherwise); and a categorical variable indicating to which general practice a patient belonged, in order to account for group differences in clinical records between the practices. The between-imputation variance was low, suggesting a moderate information loss as a result of missingness. A complete-case analysis was also performed.

### Statistical analysis

Data pre-processing included removing erroneous values (e.g. dates) and scaling BMI and HbA_1c_ to kg/m^2^ and %. Potentially erroneous BMI and HbA_1c_ values, defined as those >3 standard deviations from the mean, were deleted and treated as missing. To describe the study population, we computed means, standard deviations, frequencies and proportions. χ^2^ tests and *t* tests were used for comparison of categorical and continuous variables, respectively, for group differences between those with and without observed type 2 diabetes incidence. Kaplan–Meier curves were used to visualise the incidence of type 2 diabetes by mental health status, ethnicity and migration status.

The Cox proportional hazards model [33] was employed to investigate the associations between the ethnicity groups, migration status, mental health conditions and type 2 diabetes incidence. Different levels of adjustment were used. First, we computed hazard ratios (HRs) for the main exposure, ethnicity, adjusted for sex and age only (model 1). The second model was used to estimate the association of ethnicity and type 2 diabetes controlled for migration status, and thus included age, sex, ethnicity and migration (model 2). Interactions of migration and ethnic groups were also tested in this model to examine whether the type 2 diabetes–ethnicity associations change with migration status. Those associations with *p*<0.05 were retained based on statistical relevance. Third, we added mental health (depression and anxiety, SMI), physical health (BMI, hypertension, macrovascular comorbidities, family history of diabetes) and area-level deprivation (model 3). The interactions of SMI and migration and of SMI and ethnicity were examined in this model; those associations with *p*<0.05 were retained. As this is an observational cohort study based on routinely collected EHRs, our analyses identify associations rather than causal relationships.

The validity of proportional hazard assumptions was assessed by visualising Schoenfeld residuals and using the ‘zph()’ test in the ‘survival’ R package [34]. All analyses were conducted in R version 4.3.1 [35], using the caret, tidyr, dplyr, survival, survcompare, mice and ggplot2 packages [36–39].

### Ethics approval

All data were extracted under the terms of a signed data-sharing agreement with each primary care practice and with project-specific approval following submission of a data privacy impact assessment, approved by Lambeth Clinical Commissioning Group. Separate ethical committee approval was not required as all data were fully anonymised for the purposes of research access, and all patient-identifiable data had been removed. All methods were performed in accordance with relevant guidelines and regulations.

### Patient and public involvement

Patient and public involvement (PPI) was incorporated through engagement with members of the Maudsley Biomedical Research Centre’s PPI group at the stages of result interpretation and manuscript editing. The group included an individual with lived experience of type 2 diabetes and mental illness, an individual with migration experience, and two public members who are also NHS service users. They contributed to shaping the terminology used to describe individuals with type 2 diabetes, migration backgrounds and mental illness. Their feedback helped to ensure that the results are presented with clarity and sensitivity to the lived experiences. PPI members were not involved in data analysis or manuscript writing. Their contributions are acknowledged in this paper, and their time was reimbursed in line with national guidance.

### Results

**Fig. 3:**
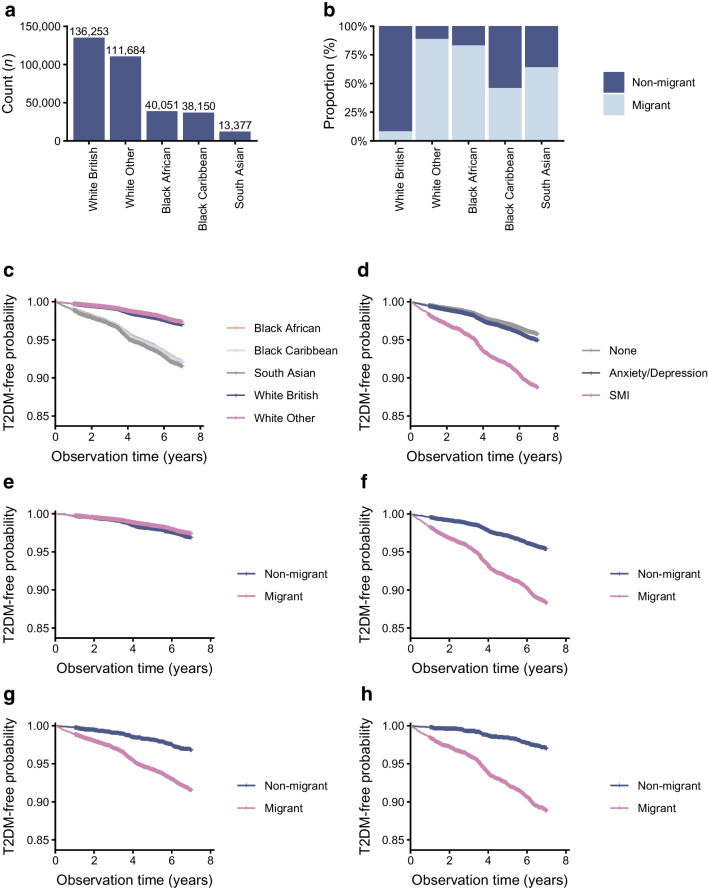
(**a**) Study population by ethnicity. (**b**) Study population by migration status. (**c**) Kaplan–Meier curves for incidence of type 2 diabetes (T2DM) by ethnicity, for the entire cohort. (**d**) Kaplan–Meier curves for T2DM incidence by mental health status, for the entire cohort. (**e**) Kaplan–Meier curves for T2DM incidence by migration status, for the Black Caribbean ethnic group. (**f**) Kaplan–Meier curves for T2DM incidence by mental health status, for the Black African ethnic group. (**g**) Kaplan–Meier curves for T2DM incidence by mental health status, for the South Asian ethnic group. (**h**) Kaplan–Meier curves for T2DM incidence by mental health status, for the White Other ethnic group

The resulting cohort included 339,515 participants, 40% were White British (*n*=136,253), 33% White Other (*n*=111,684), 12% Black African (*n*=40,051), 11% Black Caribbean (*n*=38,150) and 4% South Asian (*n*=13,377). The mean observation duration was 4.7 years, 52% were women, the mean age at study entry was 36.6 years old, and the mean BMI was 25.1 kg/m^2^. Of those who reported their country of birth, 55% were born outside the UK. The percentage of migrants differed significantly across ethnic groups: 9% of White British participants, 91% of White Other participants, 48% of Black Caribbean participants, 85% of Black African participants and 69% of South Asian participants reported being born outside of the UK (ESM Table 4). Figure 3 illustrates the differences in type 2 diabetes incidence in our cohort by country of birth status for each ethnic group, with the respective Kaplan–Meier survival curves. Those who developed type 2 diabetes during the study period (*n*=9631) were significantly older and with higher BMI (mean age 53.8 years, mean BMI 31.5 kg/m^2^) compared with those who did not develop type 2 diabetes (mean age 36.1 years, mean BMI 24.9 kg/m^2^), as shown in Table 1.

**Table 1:** Baseline characteristics of the study cohort

	Full cohort (*N*=339,515)	No type 2 diabetes (*n*=329,884; 97.2%)	Observed type 2 diabetes (*n*=9631; 2.8%)	Test statistics (*t*(*df*) or χ^2^(*df))*^a^	
					*p* value
Sex: women	175,541 (51.7)	170,801 (51.8)	4740 (49.2)	24.56(1)	<.001
Migration status^b^					
Migrant^c^	120,194 (54.4)	116,078 (54.0)	4116 (67)	403.28(1)	<.001
Non-migrant	100,751 (45.6)	98,881 (46.0)	2027 (33)		
Missing	118,570	114,925	3488		
Age at study entry (years)	36.6±13.92	36.1±13.6	53.8±13.81	125.81(339,513)	<.001
Observation duration (years)	4.74±2.46	4.77±2.46	3.48±2.09	51.21(339,513)	<.001
Type 2 diabetes incidence	9631(2.8)	–	9631(100)		
Age at type 2 diabetes diagnosis (years)	59.15±6.9	–	59.15±6.9		
Ethnicity					
White British	136,253 (40.1)	133,821 (40.6)	2432 (25.3)	4925.9(4)	<.001
White other	111,684 (32.9)	110,078 (33.4)	1606 (16.7)		
South Asian	13,377 (3.9)	12,611 (3.8)	766 (8.0)		
Black African	40,051 (11.8)	37,692 (11.4)	2359 (24.5)		
Black Caribbean	38,150 (11.2)	35,682 (10.8)	2468 (25.6)		
Area deprivation^d^					
Quintile 1 (most deprived)	60,341 (18.3)	57,804 (18)	2537 (26.8)	596.01(4)	<.001
Quintile 2	155,763 (47.1)	151,469 (47.2)	4294 (45.4)		
Quintile 3	87,588 (26.5)	85,510 (26.6)	2078 (22)		
Quintile 4	22,328 (6.8)	21,885 (6.8)	443 (4.7)		
Quintile 5 (least deprived)	4358 (1.3)	4317 (1.3)	41 (0.4)		
Missing	9137	8899	238		
BMI (kg/m^2^)^e^	25.1±5.14	24.9±4.98	31.51±6.38	−84.36(145,461)	<.001
Hypertension	21,787 (6.4)	18,532 (5.6)	3255 (33.8)	12,373.76(1)	<.001
Ischaemic heart disease	2741 (0.8)	2300 (0.7)	441 (4.6)	1760.75(1)	<.001
Heart failure	687 (0.2)	590 (0.2)	97 (1.0)	317.94(1)	<.001
Stroke	1981 (0.6)	1734 (0.5)	247 (2.6)	670.69(1)	<.001
Myocardial infarction	1277 (0.4)	1080 (0.3)	197 (2.0)	737.17(1)	<.001
Reported family history of diabetes	1121 (0.3)	1022 (0.3)	99 (1.0)	146.64(1)	<.001
Learning disability	920 (0.3)	856 (0.3)	64 (0.7)	56.81(1)	<.001
Depression	29,609 (8.7)	28,333 (8.6)	1276 (13.2)	255.29(1)	<.001
Anxiety	24,490 (7.2)	23,646 (7.2)	844 (8.8)	35.59 (1)	<.001
SMI	5332 (1.6)	4847 (1.5)	485 (5.0)	770.03(1)	<.001

Values for continuous variables are means ± SD; values for categorical variables are *n* (%)

^a^To contrast the individual characteristics of those who developed type 2 diabetes by the study end and those who did not, the *t* test (for continuous variables) and the χ^2^ test (for categorical variables) were used. The reported *t*(*df*) and χ^2^(*df*) are the statistics for the *t* tests and χ^2^ tests, with degrees of freedom (*df*); the respective *p* values are shown in the next column

^b^The percentage of migrant and non-migrant participants was calculated based on the numbers of participants with non-missing country of birth data

^c^Country of birth not UK

^d^The percentage of participants in each quintile was calculated based on the number of participants with non-missing deprivation index data

^e^The mean and SD for BMI were calculated using non-missing values only, before imputation for missing values

The results of the main association analyses are presented in Table 2 and ESM Table 5, and evidence significant differences in the type 2 diabetes risk between the ethnic groups. The ethnic disparities in type 2 diabetes risks showed no substantial attenuation when higher levels of adjustments were added, apart from the White Other group, for which results were only significant in model 1. In model 1, adjusted for age and sex only, the HR for type 2 diabetes was 1.12 (95% CI 1.05, 1.20) for White Other, 3.29 (95% CI 3.11, 3.49) for Black African, 2.92 (2.76, 3.10) for Black Caribbean, and 3.83 (3.53, 4.17) for South Asian, compared with White British.

**Table 2:** HRs and 95% CI for the longitudinal association of type 2 diabetes with ethnicity and migration status

	White British (*n*=136,253)	White Other (*n*=111,684)	Black African (*n*=40,051)	Black Caribbean (*n*=38,150)	South Asian (*n*=13,377)
Model 1 (age, sex, ethnicity)					
All (no migration adjustment)	1 (baseline)	1.12 (1.05, 1.20); *p*<0.001	3.29 (3.11, 3.49); *p*<0.001	2.92 (2.76, 3.10); *p*<0.001	3.83 (3.53, 4.17); *p*<0.001
Model 2 (age, sex, ethnicity, migration status)					
Non-migrant	1 (baseline)	0.96 (0.89, 1.04); *p*=0.3262	2.81 (2.62, 3.02); *p*<0.001	2.63 (2.47, 2.80); *p*<0.001	3.34 (3.05, 3.65); *p*<0.001
Migrant	1.33 (1.24, 1.42); *p*<0.001	1.28 (1.19, 1.37); *p*<0.001	3.73 (3.5, 3.99); *p*<0.001	3.49 (3.25, 3.75); *p*<0.001	4.43 (4.05, 4.85); *p*<0.001
Migrant vs non-migrant within ethnic group		1.33 (1.24, 1.42); *p*<0.001	1.33 (1.24, 1.42); *p*<0.001	1.33 (1.24, 1.42); *p*<0.001	1.33 (1.24, 1.42); *p*<0.001
Model 3 (fully adjusted)					
Non-migrant	1 (baseline)	0.96 (0.89, 1.04); *p*=0.3381	2.20 (2.05, 2.36); *p*<0.001	2.13 (1.99, 2.27); *p*<0.001	3.50 (3.19, 3.84); *p*<0.001
Migrant	1.29 (1.20, 1.38); *p*<0.001	1.24 (1.15, 1.33); *p*<0.001	2.83 (2.64, 3.04); *p*<0.001	2.74 (2.54, 2.95); *p*<0.001	4.51 (4.08, 4.98); *p*<0.001
Migrant vs non-migrant within ethnic group		1.29 (1.20, 1.38); *p*<0.001	1.29 (1.20, 1.38); *p*<0.001	1.29 (1.20, 1.38); *p*<0.001	1.29 (1.20, 1.38); *p*<0.001

In model 2 (with added migration variable), the ethnicity-related type 2 diabetes risks were slightly attenuated compared with model 1: 0.96 (95% CI 0.89, 1.04) for White Other, 2.81 (95% CI 2.62, 3.02) for Black African, 2.63 (95% CI 2.47, 2.80) for Black Caribbean, and 3.34 (95% CI 3.05, 3.65) for South Asian. Migration status, measured as being born outside of the UK, was associated with a 33% higher risk of type 2 diabetes (HR 1.33; 95% CI 1.24, 1.42) in model 2, and a 29% higher risk of type 2 diabetes in model 3 (HR 1.29; 95% CI 1.20, 1.38). We found no statistically significant interaction between ethnicity and migration status: being a migrant was associated with the same 33% increase in type 2 diabetes risk for all ethnic groups. Table 2 shows the combined risks of ethnicity and migration estimated using model 2.

In the fully adjusted model 3 (age, sex, migration status, area-level deprivation, BMI, macrovascular conditions, depression and anxiety, and SMI), the HRs for ethnic groups were comparable to those in model 2: 0.96 (95% CI 0.89, 1.04) for White Other, 2.20 (95% CI 2.05, 2.36) for Black African, 2.13 (95% CI 1.99, 2.27) for Black Caribbean, and 3.50 (95% CI 3.19, 3.84) for South Asian.

The depression and anxiety variable was associated with a moderate increase in type 2 diabetes risk (HR 1.15, 95% CI 1.07, 1.24). In contrast, SMI was associated with an almost double type 2 diabetes risk (HR 1.84, 95% CI 1.67, 2.03) (ESM Table 5).

We found no statistically significant interaction between ethnic groups and migration status, or between migration status and SMI, i.e. the strength of the association between ethnicity and type 2 diabetes, as well as that between SMI and type 2 diabetes, was similar for the migrant and non-migrant participants. There were no strong interactions overall, except for a modest interaction between South Asian ethnicity and SMI, suggesting a low additional type 2 diabetes risk arising from SMI for South Asians, as opposed to other ethnic groups; in South Asians, SMI was associated with an 84% increase in HR. ESM Table 6 shows the combined risks of ethnicity, migration and SMI, estimated using model 3.

The results for the complete-case analysis and those obtained using imputed data were very similar, and the HR estimates for ethnicity, migration and mental health variables did not differ by more than 15%, as shown in ESM Table 5.

## Discussion

This study used a large, representative EHR dataset from South London to examine longitudinal associations of ethnicity, migration status and mental health with incident type 2 diabetes in a multiethnic urban population. All of these exposures were associated with an increased type 2 diabetes risk.

### Ethnic inequalities in type 2 diabetes outcomes

Our study highlights ethnic inequalities in type 2 diabetes outcomes, with South Asian, Black African and Black Caribbean ethnic groups showing 2–3-fold higher type 2 diabetes risk (HR) compared with White British individuals, similar to previous UK and international evidence [5, 9]. The results for the White Other group did not differ significantly from those for the White British group in adjusted analyses.

The elevated type 2 diabetes risks for the South Asian, Black African and Black Caribbean ethnic groups persisted even after adjustment for sociodemographic, physical and mental health factors, indicating the role of unmeasured structural, environmental and behavioural determinants. The moderate attenuation of HRs for Black Caribbean, Black African and South Asian ethnic groups after adjustment for migration status, socioeconomic and health conditions suggests that these factors only partially explain the observed inequalities. The elevated type 2 diabetes risk in South Asian, Black African and Black Caribbean groups was not explained by whether individuals are first-generation migrants or were born in the UK. This finding supports the idea that structural and environmental factors tied to ethnicity (e.g. discrimination, neighbourhood deprivation, healthcare access) operate independently of migration history.

Taken together, our findings align with the UK definition of health inequalities as systematic, avoidable and unfair differences in health outcomes between population groups [40], and point towards a dominant role of structural and environmental factors such as access to care, discrimination, migration-related barriers [20] and socioeconomic disadvantage [5] in the observed ethnic disparities in type 2 diabetes rates.

### Novel contribution of this study

A key contribution of this study lies in leveraging data sources that show enhanced data collection for both self-ascribed ethnicity and country of birth, together with the rich clinical data in the EHRs. Here, we demonstrated that being a first-generation immigrant, which is a rarely examined variable due to limited availability of such information in EHRs [41], is a risk factor for incident type 2 diabetes. Previous research has predominantly focused on ethnicity as a key distinguishing factor for type 2 diabetes in multiethnic populations, while existing diabetes risk tools such as QDiabetes [42] have incorporated ethnicity and mental health variables, but not migration status.

As we hypothesised that migration and mental health conditions may be positioned on causal pathways between ethnicity and type 2 diabetes (Fig. 1), we expected an attenuation of the associations of ethnicity and type 2 diabetes incidence in the fully adjusted models compared with the basic controls for age and sex. However, contrary to our hypothesis, adjustment for migration and mental health did not substantially reduce ethnic disparities, and no statistically significant interaction was observed between ethnicity and migration. The results suggest that these exposures may constitute multiple forms of disadvantage, and align with prior reports of multiple disadvantaged statuses leading to poorer health outcomes, including increased mortality rates, through the cumulative effects of discrimination and stigmatisation [43, 44]. From a public health perspective, the largely independent effects observed across ethnicity, migration and mental health suggest that these domains may offer distinct intervention opportunities. The consistency of migration‑related effects across ethnicities suggests that post‑migration support may be relevant across diverse migrant communities, while the persistence of ethnic inequalities irrespective of migration history underscores the need for interventions that address the broader structural and environmental determinants that shape type 2 diabetes risk, including discrimination, socioeconomic disadvantage, and inequitable access to healthcare [45].

### Migration status as a risk factor for type 2 diabetes

In our cohort, within each ethnic group, being a first-generation immigrant was associated with a 29% additional type 2 diabetes risk; this result was consistent across all ethnic groups. Although the increase in risk is moderate, it applies to the majority of our study population: half of the participants were born outside the UK, and two-thirds of new type 2 diabetes cases occurred in people with migration experience. This finding aligns with the results of a recent meta-analysis [14] that showed an elevated diabetes prevalence and high heterogeneity of type 2 diabetes risks among international immigrants in North America, Italy, Sweden, the Netherlands, Australia and Israel.

The persistence of the migration effect after extensive adjustment suggests that migration-related factors may contribute to metabolic health. As all participants were type 2 diabetes-free at baseline and developed diabetes during follow-up, with an average time to type 2 diabetes diagnosis of 3.2 years (SD 2.1), and more than 50% had been registered for at least 2 years at the baseline (ESM Table 7), it may be hypothesised that post-migration exposures play a substantial role in type 2 diabetes development. For instance, 44% of migrants in our sample reported a primary language other than English, compared with only 1% of those born in the UK, highlighting potential barriers to care and prevention [20]. Other contributing factors may include acculturation stress, reduced healthcare access, dietary and lifestyle changes, and language barriers [13]. Although such mechanisms are hypothesised, their impact on type 2 diabetes risk remains largely unquantified due to the limited migration‑related data [41].

Our results do not support a ‘healthy migrant’ hypothesis. Prior studies showed that first-generation migrants to the USA exhibited better health outcomes than those of the same ethnicity born in the USA, although this diminished with duration of stay [16]. A more complicated picture was found for South Asian residents of Canada [6]: type 2 diabetes prevalence among first-generation migrants was lower for some countries of birth and higher for others compared with South Asians born in Canada. This underlines the heterogeneity of the multiethnic migrant populations and suggests that migration type may influence risk. For example, forced migration due to natural disasters, political unrest or armed conflicts can impose a financial, emotional and physical burden on migrants’ lives, and affect mental health and cardiometabolic outcomes [41]. Given the rapidly growing scale of migration globally [46], further research should explore these links to guide national healthcare services responses [47]. Adjusting for pre-migration experiences would help to disentangle the pre- and post-migration factors associated with type 2 diabetes development, although we acknowledge that recording migration reasons in EHRs could pose ethical challenges [41].

Summing up, our country‑of‑birth measure reflects only an aggregate view of migration’s impact on type 2 diabetes risk. Given the heterogeneity within migrant populations arising from migration pathways, pre‑migration exposures, and duration of residence, there may be meaningful variation in type 2 diabetes risk across migrant subgroups, which future studies should explore in more detail. Our findings thus also advocate for systematic recording of migration‑related data in primary care, including country of birth, to enable clearer identification of the mechanisms through which migration may influence type 2 diabetes risk.

### Mental health comorbidities as risk factors for type 2 diabetes

Common mental illnesses (depression and anxiety) and SMI (schizophrenia, bipolar disorder and other non-organic psychoses) were each associated with higher type 2 diabetes risk across ethnic groups, with the exception of a reduced combined effect of SMI and South Asian ethnicity. Depression and anxiety were linked to a modest increase in risk, while SMI nearly doubled the type 2 diabetes risk. In our cohort, depression/anxiety and SMI conferred comparable HRs across the various ethnic and migrant groups, which may imply that mental health‑informed diabetes prevention strategies could be relevant across ethnic and migrant groups.

The findings are consistent with prior evidence suggesting that both common mental illnesses and SMI increase diabetes risk through behavioural, biological and healthcare access pathways [13, 25, 48]. People with mental illness may encounter barriers to preventive care, leading to delayed detection and management of type 2 diabetes [49]; reported difficulties in distinguishing early metabolic symptoms from mental health-related symptoms [50] may further contribute to diagnostic delays.

### Strengths and limitations

The study comprised a large multiethnic cohort, and the use of longitudinal EHRs strengthens the reliability of findings. More than 98% of people in the UK are registered with a primary healthcare centre, which ensures population representativeness of the covered area. The study covers the most recent pre-COVID-19 period (thus avoiding pandemic-related disruptions to help-seeking and EHR recording), making the results generalisable to current clinical practice. Inclusion of diverse risk factors such as deprivation, migration status and minority ethnic group enabled examination of multiple vulnerability domains.

Several limitations should be acknowledged. A key limitation was the use of country of birth as a crude measure of migration, which represented a heterogeneous group of first-generation migrants. We lacked data on migration reasons, preventing separation of pre- and post-migration influences and different migrant generations. Also, some ethnic subgroups were merged due to small numbers, obscuring within-group differences. Future research should collect more granular data to investigate the factors and mechanisms behind the migration-related differences in type 2 diabetes outcomes. We did not collect data on mental health conditions such as post-traumatic stress disorder and eating disorders, which have been linked to type 2 diabetes and the psychological stress of migration [13]. Future research could explore the contribution of these factors to the incidence of type 2 diabetes within ethnically diverse and migrant populations. Second, the study inclusion criterion of at least 12 months’ consecutive primary care registration may have systematically excluded certain high-risk groups, particularly recent migrants and people of lower socioeconomic status, which could have biased the observed migration effect downwards. Missing data required imputation, which could have introduced bias. Finally, although the temporal order of exposure and outcome may indicate potential causal associations, this study remains observational and is subject to residual confounding and reverse causation. The reported HRs should thus be interpreted as measures of association between exposures and incident type 2 diabetes among individuals with comparable measured characteristics. More elaborate models, including structural equation models or use of mediation analysis, are required to determine causality.

### Conclusion

In our cohort, ethnicity, migration and mental illness each increased type 2 diabetes risk in urban multiethnic populations, with migration conferring additional risk within ethnic groups. There was no evidence of strong interacting effects, suggesting that each domain represents a distinct intervention opportunity. Our finding that migration‑related stressors did not appear to vary substantially across ethnic groups suggests that support addressing post‑migration challenges such as language assistance, service navigation or psychosocial support could be relevant across diverse migrant communities. Likewise, the persistence of ethnic inequalities irrespective of migration history points to the need for interventions that address broader structural and environmental factors linked to ethnicity, including discrimination, socioeconomic factors and inequitable access to healthcare, alongside service‑level and behavioural approaches.

## Supplementary Information

Below is the link to the electronic supplementary material.

ESM Tables (PDF 432 KB)

## Data Availability

The datasets generated and/or analysed during the current study are not publicly available due to restrictions on use of the data for the approved researchers and projects only. The data are therefore available from the authors on request, subject to obtaining project-specific data access approvals from the NHS South East London Integrated Care System (SE London ICS [51]) and in accordance with recent guidelines from the Lambeth DataNet [52].

## Abbreviations

EHR: Electronic health record
SMI: Severe mental illness
