# Supplementary material for: Inequalities in type 2 diabetes incidence in a multiethnic population: a cohort study investigating the impact of ethnicity, migration and mental health comorbidities
Source: Diabetologia. 2026 Apr 22;69(8):2240–52. doi: 10.1007/s00125-026-06740-3 (PMC13310228; doi:10.1007/s00125-026-06740-3)
Supplement: Supplementary file 1 — ESM Tables (PDF 432 KB) [file 125_2026_6740_MOESM1_ESM.pdf]

Inequalities in type 2 diabetes incidence in a multiethnic population: a cohort study investigating the impact of ethnicity, migration and mental health comorbidities

Diana Shamsutdinova*[1,2], Daniel Stahl[1,2], Jayati Das-Munshi[3,4,5,6]

Electronic supplementary materials (ESM)

Contents

Electronic supplementary materials (ESM) 1

ESM Table 1. Clinical codes (UK Read Codes) for the extraction of clinical mental health diagnoses. 2

ESM Table 2. Defining categories for ethnicity variable in this study 6

ESM Table 3. Missing values 7

ESM Table 4. Study population by ethnicity, SMI and Birth Country 8

ESM Table 5. Estimated coefficients of the CoxPH models (Model 1, Model 2, Model 3) for the outcome of Type 2 Diabetes Mellitus (T2DM) incidence. 9

ESM Table 6. Estimated hazard ratios for T2DM incidence by ethnic group, migration status and SMI diagnosis. 10

ESM Table 7. Share of participants by the duration of available medical history by migration status. 10

ESM Table 1. Clinical codes (UK Read Codes) for the extraction of clinical mental health diagnoses.

**Term**	**Read Code**
Type 1 Diabetes	C10E%
Type 2 Diabetes	C10F%, C109%
HBA_{1c}	42W5 (IFFC), 42W4 (DCCT)
Fasting plasma glucose	44g1
Secondary Diabetes	C10H%, C10B, C10N%, C10G%
Other types of diabetes	C10A, C10C, C10D, C1A%, C10M%, C326%, A3A2, PH3y5, M212, C135, PKyP, Q441, C10FS, C150%, C3500, C10N1, C370%, PKyF, C1zy4, PH3yA, PKy93, PKy1
Pancreatic disease/surgery	J670%, J671%, J6710, J6711, J67y6, 9b8G
Diabetes in pregnancy	L1808, L1809
Diabetes emergencies	C10EM, C109K
Severe mental illness:<br>Psychosis, schizophrenia, bipolar affective disease codes	E10..% , E110.% , E111.% , E1124 , E1134 , E114.- E117z , E11y.% (excluding E11y2) , E11z. , E11z0 , E11zz , E12..% , E13..% (excluding E135.) , E2122 , Eu2..% , Eu30.% , Eu31.% , Eu323 , Eu328 , Eu333 , Eu32A , Eu329
Depression	E0013 , E0021 , E112.% , E113.% , E118. , E11y2 , E11z2 , E130. , E135. , E2003 , E291. , E2B.. , E2B1. , Eu204 , Eu251 , Eu32.% (excluding Eu32A , Eu32B , Eu329) , Eu33.% , Eu341 , Eu412
Anxiety	E200%
Polycystic ovarian syndrome	C165
Chronic kidney disease	1Z10.00, 1Z17.00, 1Z17.11, 1Z18.00, 1Z11.00, 1Z19.00, 1Z19.11, 1Z1A.00, 1Z1A.11, 1Z12.00, 1Z15.00, 1Z16.00, 1Z1B.00, 1Z1B.11, 1Z1C.00, 1Z1C.11, 1Z1D.00, 1Z1D.11, 1Z1E.00, 1Z1E.11, 1Z1F.00, 1Z1F.11, 1Z1G.00, 1Z13.00, 1Z1H.00, 1Z1J.00, 1Z1J.11, 1Z14.00, 1Z1K.00, 1Z1L.00, 1Z1L.11
Diabetic Retinopathy	2BBF.00, F420.00, F420z00, 2BBP.00, 2BBQ.00, F420000, 2BBR.00, 2BBS.00, F420200, F420500, F420600, F420800, 2BBT.00, 2BBV.00, 2BBo.00, F420100, F420700, 2BBk.00, 2BBl.00, 7276.
Diabetic neuropathy	C106.00, C106.11, C106.12, C106.13, C106000, C106100, C106y00, C106z00, C108200, C108211, C108212, C108B00, C108C00, C108J00, C108J11, C108J12, C109200, C109211, C109212, C109A00, C109A11, C109B00, C109B11, C109H00, C109H11, C109H12, C10E200, C10EB00, C10EC00, C10EC11, C10EJ00, C10EQ00, C10F200, C10F211, C10FA00,

	C10FA11, C10FB00, C10FB11, C10FH00, C10FR00, F171100, F345000, F35z000, F372.00, F372.11, F372.12, F372000, F372100, F372200, F381300, F381311, F3y0.00, M271100, N030100
Amputation	2G42. , 2G44. , 2G46., 2G43. , 2G45. , 2G47
Other eye complications	C105.00, C105000, C105100, C105y00, C105z00, C108100, C108F00, C108F11, C109100, C109111, C109112, C109E00, C109E11, C109E12, C10E100, C10E112, C10EF00, C10F100, C10FE00, C10FE11, F464000, 2BBL.00, 2BBW.00, 2BBX.00, C10EP00, C10EP11, C10FQ00, F420300, F420400
Diabetic foot ulcer	2G5L, 2G55, 2G5H, 2G54
Ischaemic Heart Disease\Coronary Heart Disease	G3, G30%, G31%, G32, G33%, G34%, G35%, G38%, G39%, G3y%, G3z, Gyu3%
Atrial fibrillation & flutter	G573%
Heart failure	G58..% , G1yz1 , 662f.-662i.
Stroke/Transient Ischemic Attack	G61%, G63y0,G63y1, G64%, G66%, G6760, G6W, G6X, Gyu62, Gyu63, Gyu64, Gyu65,Gyu66, Gyu6F, Gyu6G, ZV12D, Fyu55, G65%
Hypertension	G2, G20%, G24%, G25%, G26, G28, G2y, G2z, Gyu2, Gyu20
Peripheral vascular disease	G7310, G732, G7320, G7321, G7322, G7323, G7324, G733, G734, G735, G73y, G73y0, G73y1, G73z, G73z0, G73zz
Peripheral arterial disease	G73, G734, G73y, G73z%, Gyu74,
Abdominal aortic aneurysm	G71%
Myocardial infarction	G30%, G310%
Angina stable	G33%
Angina unstable	G311%

Other atherosclerotic disease	F421100, F421112, G342.00, G5y2.00, G70..00, G70..11, G70y.00, G70z.00
Cardiac valve disorders	791..00, 7910., 7910.11, 7910.12, 7910000, 7910100, 7910200, 7910211, 7910212, 7910213, 7910214, 7910300, 7910400, 7910y00, 7910z00, 7911., 7911.11, 7911.12, 7911000, 7911100, 7911200, 7911300, 7911400, 7911y00, 7911z00, 7912., 7912.11, 7912.12, 7912000, 7912100, 7912200, 7912300, 7912y00, 7912z00, 7913., 7913.11, 7913.12, 7913000, 7913100, 7913200, 7913300, 7913400, 7913y00, 7913z00, 7914., 7914.11, 7914000, 7914100, 7914200, 7914211, 7914212, 7914300, 7914400, 7914y00, 7914z00, 7915., 7915000, 7915100, 7915200, 7915300, 7915y00, 7916.11, 7916000, 7916100, 7916200, 7916300, 7916z11, 7917.11, 7917000, 7917100, 7917300, 7918000, 7919.11, 7919000, 7919100, 7919300, 7919400, 791A200, 791A300, A932.11, G11..00, G11..11, G110.00, G110.11, G111.00, G111.11, G111.12, G112.00, G112.12, G112.13, G113.00, G114.00, G11z.00, G12..00, G120.00, G121.00, G121.11, G121.12, G122.00, G12z.00, G13..00, G130.00, G131.00, G131.13, G131.14, G132.00, G132.12, G132.13, G133.00, G133.11, G133.12, G13y.00, G13z.00, G140.00, G140000, G140100, G140111, G140112, G140200, G14021X, G14021Y, G140300, G140400, G140412, G140413, G140500, G140514, G140z00, G141.00, G141000, G141100, G141z00, G54..11, G540.00, G540.12, G540.14, G540.15, G540.16, G540000, G540100, G540200, G540300, G540z00, G541.00, G541000, G541011, G541012, G541100, G541200, G541211, G541212, G541300, G541400, G541500, G541600, G541z00, G542.00, G542000, G542011, G542012, G542100, G542200, G542X00, G542z00, G543.00, G543000, G543011, G543012, G543100, G543200, G543213, G543215, G543300, G543311, G543400, G543z00, G544.00, G544000, G544100, G544200, G544X00, G54z000, G54z013, G54z014, G54z100, G54z500, Gyu1000, Gyu1100, Gyu5500, Gyu5600, Gyu5800, Gyu5A00, P602.00, P602z00, P61..00, P611.00, P62..00, P63..00, P64..00, P640.00, P641.00, P64z.00, P65..00, P650.00, P66..00, P6W..00, P6X..00, P6y0.00, P6y2.00, P712.13, P722400
Venous thromboembolism	G801.00, G801.12, G801600, G801700, G801B00, G801C00, G801E00, G801F00, G801z00, F05..00, F051.00, F051z00, F053.00, F053000, F053100, F05z.00, G676.00, G801.11, G801.13, G801D00, G80y.11, G80y400, G80y500, G80y600, G80y700, G80y800, G81..00, G820.00, G820.11, G822.00, G822000, Gyu8000, J420200, L413.00, L413.11, L413000, L413100, L413z00, L414.00, L414.11, L414000, L414200, L414z00, SP12200, 7A09300, 7A09311, 7A0A100, G401.00, G401.12, G401000, G401100, L096400, L43..00, L43..11, L430.00,

	L431.00, L431100, L432.00, L43z.00, L43z100, L43z400, L43zz00
Cardiomyopathy	G551.00, G554300, Gyu5M00

ESM Table 2. Defining categories for ethnicity variable in this study

Collected data	*Grouping for this study*	*included/ excluded*
African	Black African	**included**
Any other ethnic group	Other Ethnic Group	excluded
Arab	Other Ethnic Group	excluded
Bangladeshi	South Asian	**included**
British	White British	**included**
Caribbean	Black Caribbean	**included**
Chinese	Asian Other	excluded
Gypsy or Irish Traveller	White Other	**included**
Indian	South Asian	**included**
Irish	White Other	**included**
Not Stated/Refused	NA	excluded
Other Asian	Asian Other	excluded
Other Black	Black Caribbean	**included**
Other Mixed	Other Ethnic Group	excluded
Other White	White Other	**included**
Pakistani	South Asian	**included**
Unknown	NA	excluded
White and Asian	South Asian	**included**
White and Black African	Black African	**included**
White and Black Caribbean	Black Caribbean	**included**

ESM Table 3. Missing values

	Missing %, All	*Missing %, SMI*
Age	0	0
Physical and Mental Health Comorbidities [(1)]	0	0
Deprivation index	2.7%	3.3%
Migrant (Country of birth not GBR)	34.9%	31.3%
Ethnicity	0	0
Language	16.2%	14.1%
Baseline BMI	57.2%	43.6%
Baseline HBA_{1c}	89.6%	80.6%
Any value during observation BMI [(2)]	30.5%	8.9%
Any value during observation HBA_{1c} [(2)]	57.5%	25.8%

[(1)] Comorbidities are all physical and mental health diagnoses that were established from the clinical codes. As the assumption that a diagnosis is present if diagnosed and vice versa, no missing values are generated. Note, 0 missingness rather reflects this assumption and does not exclude from biases in the data from undiagnosed or uncoded conditions, or errors in EHR.

[(2)] Refers to the percentages of participants for whom no measurements were available at any point during the 7 years of observation.

ESM Table 4. Study population by ethnicity, SMI and Birth Country

		ALL		SMI		non-SMI	
Ethnicity	**Migrant or non-Migrant (Country of Birth Not GBR)**	*N*	%	*N*	%	*N*	%
Black African	Non-migrant	**4100**	**15.5%**	172	27.9%	3928	15.2%
Black African	Migrant	**22,402**	**84.5%**	444	72.1%	21,958	84.8%
Black African	No record	**13,549**		214		13,335	
Black Caribbean	Non-migrant	**11,820**	**52.0%**	569	62.1%	11,251	51.6%
Black Caribbean	Migrant	**10,892**	**48.0%**	347	37.9%	10,545	48.4%
Black Caribbean	No record	**15,438**		446		14,992	
South Asian	Non-migrant	**2777**	**31.3%**	57	43.5%	2720	31.1%
South Asian	Migrant	**6103**	**68.7%**	74	56.5%	6029	68.9%
South Asian	No record	**4497**		55		4442	
White British	Non-migrant	**74,551**	**91.3%**	1206	92.6%	73,345	91.3%
White British	Migrant	**7087**	**8.7%**	97	7.4%	6990	8.7%
White British	No record	**54,615**		706		53,909	
White Other	Non-migrant	**7582**	**9.3%**	155	22.2%	7427	9.2%
White Other	Migrant	**73,710**	**90.7%**	542	77.8%	73,168	90.8%
White Other	No record	**30,392**		248		30,144	

ESM Table 5. Estimated coefficients of the CoxPH models (Model 1, Model 2, Model 3) for the outcome of Type 2 Diabetes Mellitus (T2DM) incidence.

		complete cases	*imputed*
Variable Name	**Model***	**HR (95% CI), *p*-value**	**HR (95% CI), *p*-value**
Age (per 10y)	**1**	1.74(1.67,1.8); *p*<0.001	1.74(1.67,1.8); *p*<0.001
Sex	**1**	0.92(0.88,0.96); *p*<0.001	0.92(0.88,0.96); *p*<0.001
Black African	**1**	3.29(3.11,3.49); *p*<0.001	3.29(3.11,3.49); *p*<0.001
Black Caribbean	**1**	2.92(2.76,3.09); *p*<0.001	2.92(2.76,3.09); *p*<0.001
South Asian	**1**	3.83(3.53,4.16); *p*<0.001	3.83(3.53,4.16); *p*<0.001
White Other	**1**	1.12(1.05,1.2); *p*<0.001	1.12(1.05,1.2); *p*<0.001
Age (per 10y)	**2**	1.76(1.69,1.85); *p*<0.001	1.73(1.67,1.8); *p*<0.001
Sex	**2**	0.91(0.87,0.96); *p*<0.001	0.92(0.88,0.95); *p*<0.001
Black African	**2**	2.93(2.66,3.23); *p*<0.001	2.81(2.63,3.01); *p*<0.001
Black Caribbean	**2**	2.70(2.48,2.94); *p*<0.001	2.63(2.47,2.8); *p*<0.001
South Asian	**2**	3.37(3,3.78); *p*<0.001	3.34(3.06,3.65); *p*<0.001
White Other	**2**	1.01(0.92,1.11); *p*= 0.8399	0.96(0.9,1.04); *p*= 0.3145
Migrant (Born outside of UK)	**2**	1.23(1.14,1.32); *p*<0.001	1.33(1.24,1.42); *p*<0.001
Age (per 10y)	**3**	1.63(1.52,1.76); *p*<0.001	1.53(1.47,1.58); *p*<0.001
Sex	**3**	0.72(0.67,0.78); *p*<0.001	0.82(0.79,0.86); *p*<0.001
Black African	**3**	2.08(1.78,2.43); *p*<0.001	2.20(2.05,2.36); *p*<0.001
Black Caribbean	**3**	2.04(1.77,2.34); *p*<0.001	2.13(1.99,2.27); *p*<0.001
South Asian	**3**	3.97(3.31,4.77); *p*<0.001	3.50(3.19,3.83); *p*<0.001
White Other	**3**	1.01(0.87,1.18); *p* = 0.8992	0.96(0.89,1.04); *p*= 0.3288
Migrant (Born outside of UK)	**3**	1.25(1.11,1.41); *p*<0.001	1.29(1.2,1.38); *p*<0.001
Area-level deprivation (decile)	**3**	0.94(0.92,0.97); *p*<0.001	0.94(0.93,0.96); *p*<0.001
Depression & Anxiety	**3**	1.17(1.02,1.34); *p*= 0.0204	1.15(1.07,1.24); *p*<0.001
SMI - Severe Mental Illness	**3**	1.65(1.42,1.92); *p*<0.001	1.84(1.67,2.03); *p*<0.001
BMI (per 5 units)	**3**	1.60(1.56,1.65); *p*<0.001	1.58(1.55,1.61); *p*<0.001
Family history of diabetes	**3**	2.25(1.51,3.36); *p*<0.001	3.01(2.44,3.72); *p*<0.001
Hypertension	**3**	1.51(1.38,1.66); *p*<0.001	1.47(1.4,1.55); *p*<0.001
Macro-vascular comorbidity	**3**	1.09(1.04,1.15); *p*<0.001	1.12(1.09,1.16); *p*<0.001
South Asian x SMI cross-term	**3**	0.57(0.29,1.14); *p*= 0.1084	0.56(0.34,0.91); *p*= 0.019

*Model 1: age, sex, ethnicity. Model 1 had no missing values; therefore, the results are identical.
Model 2: age, sex, ethnicity, migration status
Model 3: age, sex, ethnicity, migration status, physical (BMI, number of macro-vascular diseases, hypertension) and mental health comorbidities (depression and anxiety, severe mental illness), and area-level deprivation.

ESM Table 6. Estimated hazard ratios for T2DM incidence by ethnic group, migration status and SMI diagnosis.

The hazard ratios are with respect to White British, non-migrant, non-SMI group. These are HRs adjusted for age, sex, area-level deprivation, BMI, number of macro-vascular diseases, hypertension, depression and anxiety.

	White British	*White Other*	*Black African*	*Black Caribbean*	*South Asian*
Model 3 (fully adjusted)	HR (95% CI), p-value	HR (95% CI), p-value	HR (95% CI), p-value	HR (95% CI), p-value	HR (95% CI), p-value
Non-Migrant (non-SMI)	*1 (baseline)*	0.96(0.89, 1.04); P= 0.3381	2.2(2.05, 2.36); *p*<0.001	2.13(1.99, 2.27); *p*<0.001	3.5(3.19, 3.84); *p*<0.001
Non-Migrant & SMI	1.84(1.67, 2.03); *p*<0.001	1.78(1.57, 2.01); *p*<0.001	4.05(3.6, 4.56); *p*<0.001	3.92(3.5, 4.39); *p*<0.001	3.61(2.23, 5.86); *p*<0.001
Migrant (non-SMI)	1.29(1.2, 1.38); *p*<0.001	1.24(1.15, 1.33); *p*<0.001	2.83(2.64, 3.04); *p*<0.001	2.74(2.54, 2.95); *p*<0.001	4.51(4.08, 4.98); *p*<0.001
Migrant & SMI	2.37(2.1, 2.69); *p*<0.001	2.29(2.02, 2.59); *p*<0.001	5.22(4.62, 5.9); *p*<0.001	5.05(4.47, 5.7); *p*<0.001	4.65(2.86, 7.58); *p*<0.001
HR for SMI vs non-SMI within ethnic group	1.84(1.67, 2.03); *p*<0.001	1.84(1.67, 2.03); *p*<0.001	1.84(1.67, 2.03); *p*<0.001	1.84(1.67, 2.03); *p*<0.001	**1.03(0.63, 1.68); p=0.8959**

ESM Table 7. Share of participants by the duration of available medical history by migration status.

Share of participants by the duration of available medical history

	**Born in UK**	**Migrant**	**All**
≤ 2 years	40.80%	48.90%	48.60%
2 to 5 years	15.00%	18.60%	12.70%
>5 years	44.20%	32.50%	38.70%
total	100.00%	100.00%	100.00%
≤ 5 years	55.80%	67.50%	61.30%
> 5 years	44.20%	32.50%	38.70%
total	100.00%	100.00%	100.00%
